# Estimating Bridge Natural Frequencies Based on Modal Analysis of Vehicle–Bridge Synchronized Vibration Data

**DOI:** 10.3390/s24041060

**Published:** 2024-02-06

**Authors:** Eugene Mudahemuka, Masatatsu Miyagi, Ryota Shin, Naoki Kaneko, Yukihiko Okada, Kyosuke Yamamoto

**Affiliations:** 1 Graduate School of Science and Technology, University of Tsukuba, 1-1-1 Tennodai, Tsukuba 305-8577, Japan; s2330208@u.tsukuba.ac.jp (E.M.); s2320891@u.tsukuba.ac.jp (M.M.); shin.ryota.sp@alumni.tsukuba.ac.jp (R.S.); s2220837@u.tsukuba.ac.jp (N.K.); 2 Center for Artificial Intelligence Research/Institute of Systems and Information Engineering, University of Tsukuba, 1-1-1 Tennodai, Tsukuba 305-8577, Japan; okayu@sk.tsukuba.ac.jp

**Keywords:** vibration-based structural health monitoring, modal analysis, vehicle–bridge interaction system

## Abstract

This paper presents a method for accurately estimating the natural frequencies of bridges by simultaneously measuring the acceleration vibration data of vehicles and bridges and applying modal analysis theory. Vibration sensors synchronized with GPS timing were installed on both vehicles and bridges, achieving stable and high-precision time synchronization. This enabled the computation of the bridge’s Frequency Response Functions (FRFs) for each mode, leading to a refined estimation of natural frequencies. The validity of the theory was confirmed through numerical simulations and experimental tests. The simulations confirmed its effectiveness, and similar trends were observed in actual bridge measurements. Consequently, this method significantly enhances the feasibility of bridge health monitoring systems. The proposed method is suitable for road bridges with spans ranging from short- to medium-span length, where the vehicle is capable of exciting the bridge.

## 1. Introduction

The development of vibration-based Structural Health Monitoring (SHM) schemes could benefit bridge inspections, offering a cost-effective alternative to conventional methods. However, the adoption of these schemes in bridge inspections is not universally acknowledged at present, partly because bridge inspection methods must achieve accuracy comparable to close visual inspection in some countries.

Although the adoption of SHM is not yet widespread, its development has significantly changed the maintenance, management, and decision-making process related to bridge engineering. Vibration measurements are quick and cost-effective using vibration-based SHM, making it an effective tool for bridge structural health monitoring [1,2]. Data acquisition and processing techniques for SHM have been developed(refined), as a result the data inaccuracy and redundancy is reduced [3,4].

Existing vibration-based SHM systems for bridge inspections primarily focus on measuring the vibrations of bridge structures [5,6]. The Fourier spectrum of free vibrations enables accurate estimation of low-order natural frequencies of a bridge [7,8,9]. This approach is instrumental in assessing the overall structural integrity, leveraging the dominant low-frequency modes that are easily excited and observed in typical bridge structures.

Bridge damage can be modeled as changes in stiffness, and these changes are detectable through variations in natural frequencies. However, many instances of bridge damage are localized [10,11], and low-order vibration indicators have limited sensitivity to such localized damage [12]. In addition, environmental factors such as changes in temperature and noise affect the bridge’s natural frequency estimation accuracy significantly [13,14,15]. Hence it is necessary to use high-order vibration indicators for more precise detection of localized structural damage, as these high-order modes are more responsive to changes in localized stiffness.

Estimating high-order modes presents technical challenges. Higher-order modes are less likely to be excited in free decay vibrations, making their observation difficult. Additionally, these modes are often obscured by noise, compromising the accuracy of their estimation. In medium-span bridges, traffic-induced vibrations may predominantly excite high-order modes [16]. However, methods to estimate these modes from transient responses have not been yet developed.

The estimation of high-order modes in bridge structures necessitates multi-point measurements. Methods like Frequency Domain Decomposition (FDD) [17,18] and Stochastic Subspace Identification (SSI) [19] are used to estimate the natural frequencies and mode shapes of bridges from multi-point measured traffic vibrations. However, these schemes assume external forces as white noise, which is a questionable assumption for traffic-induced vibrations and thus may lack reliability. Consequently, the estimated natural frequencies may not always be accurate and could merely represent predominant vibration frequencies.

A classical method for high-precision system identification from transient response data involves the use of Frequency Response Functions (FRFs). In controlled excitation systems, measuring input and output allows for the calculation of the FRF in the frequency domain by taking their ratio. Peaks in the FRF indicate the system’s natural frequencies. Additionally, the FRF allows the determination of mode shapes through the utilization of multi-point measurements of input and output.

In a Vehicle–Bridge Interaction (VBI) system [20], the input can be considered as vehicle vibrations, and the output as bridge vibrations. Nevertheless, to adopt FRF-based strategies for bridge inspections, it is necessary to establish identification strategies that take into account the motion of traffic loads. Furthermore, the development of reliable methods for time synchronization between separate measurement systems installed on vehicles and bridges is essential. This approach has the potential to enhance the accuracy and applicability of SHM in evaluating the structural health of bridges under real-world conditions.

To account for the movement of traffic loads in VBI systems [21], it is established that decomposing the equations of motion of the bridge into modal components is an effective approach. By applying a mode shape function as a window function to the traffic load in the equation of motion of each modal order, the use of modal loads as inputs becomes feasible. This procedure enables the calculation of the FRF for each modal order. However, the modal decomposition of bridge vibration data necessitates multi-point measurements on the bridge, and the creation of window functions requires knowledge of the vehicle’s relative position [22,23,24].

Nagayama and Spencer [25] have developed a wireless sensor network (WSN) system. It has been demonstrated that WSNs can achieve time synchronization, even in large-scale bridge networks, using wireless communication. However, this system was designed for multi-point measurements on bridges and does not consider time synchronization between vehicles and bridges. Furthermore, WSNs may not be ideally suited for simultaneous vibration measurements of vehicles and bridges, as they can experience data loss during vehicle passages depending on the wireless frequency band used. These limitations highlight the need for further development in synchronization technology to effectively implement VBI systems in bridge SHM.

On the other hand, the increasing number of satellites has improved the positioning accuracy of Global Navigation Satellite Systems (GNSS). GNSS devices mounted on a vehicle enable the calculation of window functions for VBI analysis [26]. Despite the improvements in GNSS technology for time synchronization, GNSS device utilization still faces difficulties such as signal blockages and vulnerability to jamming and spoofing activities [27]. Signal blockages can affect the accuracy of GNSS devices which can lead to potential disruptions in time synchronization and impact the accuracy of natural frequency estimation; a clock error of up to sub-20 microseconds can be observed during a complete blockage on the road but still meets the desired requirements of most vehicular communications [27,28]. Despite the challenges associated with their utilization, GNSS can be considered highly accurate clocks, measuring the signal propagation time from multiple satellites to the GNSS device. This capability allows for time synchronization between independent sensor systems on vehicles and bridges through GNSS.

Therefore, the purpose of this study is to propose an FRF-based simultaneous monitoring method for vehicles and bridges. This method aims to accurately estimate the natural frequencies of bridges, particularly focusing on high-order modal frequencies. By utilizing GNSS devices for time synchronization, this approach addresses the challenges in detecting high-order modes in bridge SHM. This method demonstrates a technique for simultaneously measuring vehicle and bridge vibration data and estimating the bridge FRF. Methods focusing on the estimation of FRFs have been investigated in the past. Yang et al. [29,30] estimated vehicle FRFs and bridge FRFs from vehicle vibration data, showing the potential for application in bridge health assessment. On the other hand, the proposed method in this article requires the additional effort of installing sensors not only on the vehicle but also on the bridge. However, GPS time-synchronized sensors significantly reduce the effort of simultaneous measurement, eliminating the cost advantage of a vehicle-vibration-only approach. The advancement in acceleration data measurement device technology enables the practice and development of monitoring methods that have been previously avoided. The effectiveness of the proposed method is validated through numerical simulations and field experiments on an actual steel bridge and a 10-ton vehicle, demonstrating its potential to enhance the precision and reliability of SHM practices for bridge inspections.

## 2. Basis Theory

In this study, a simultaneous monitoring method is proposed to accurately estimate the bridge’s natural frequency based on the frequency response functions (FRFs) of the bridge. This method exploits the possibility of measuring both vehicle and bridge vibration data simultaneously to increase the accuracy of estimating the higher modes of bridge vibration. The vehicle’s position as it crosses the bridge is determined by using GPS temporal and spatial synchronization.

### 2.1. Vehicle Bridge Interaction System

#### 2.1.1. Bridge System

**Figure 1:**
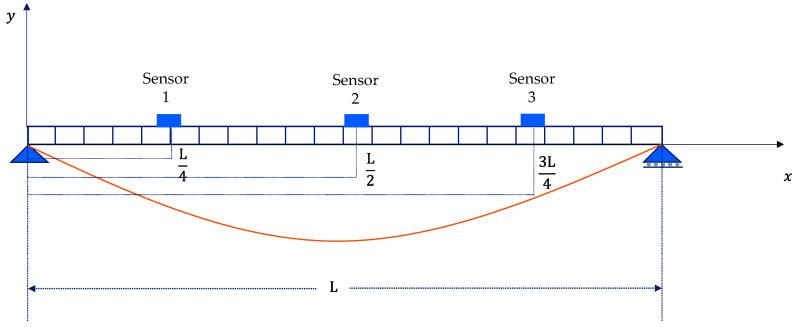
The bridge system model with sensor locations.

For numerical simulation of the VBI system, the bridge system is modeled as a simply supported Euler–Bernoulli beam Figure 1. The general equation of motion for this model is given by Equation (1) [31,32].

(1) $$\rho A\ddot{y}\left(x,t\right)+\frac{\partial^{2}}{\partial x^{2}}EI\frac{\partial^{2}}{\partial x^{2}}y\left(x,t\right)=-\sum_{i=1}^{n}\delta \left(x-x_{i}\left(t\right)\right)\left\{m_{si}\left(g-{\ddot{z}}_{si}\left(t\right)\right)+m_{ui}\left(g-{\ddot{z}}_{ui}\left(t\right)\right)\right\}$$

where x is the position along the bridge, t is time, ρ is the mass density of the bridge, A is the cross-sectional area, E is the Young’s modulus, I is the inertia moment, $m_{si}$ is the mass of $i^{th}$ sprung mass of the vehicle body, $m_{ui}$ is i-th unsprung mass of the vehicle, g is the gravitational acceleration, ${\ddot{z}}_{si}\left(t\right)$ is the vehicle’s sprung-mass vertical acceleration, ${\ddot{z}}_{ui}\left(t\right)$ is the vehicle’s unsprung-mass vertical acceleration, $y\left(x,t\right)$ is the bridge’s vertical deflection, and $\ddot{y}\left(x,t\right)$ are the bridge’s vertical acceleration vibrations. Applying the Galerkin method simplifies Equation (1) to Equation (2) as follows [33]:

(2) $$M_{b}\ddot{y}\left(t\right)+C_{b}\dot{y}\left(t\right)+K_{b}y\left(t\right)=f_{b}\left(t\right)$$

where $M_{b}$ is the mass matrix, $C_{b}$ is the damping matrix, $K_{b}$ is the stiffness matrix for the bridge system, and $f_{b}\left(t\right)$ is the contact force from the vehicle.

#### 2.1.2. Vehicle System

**Figure 2:**
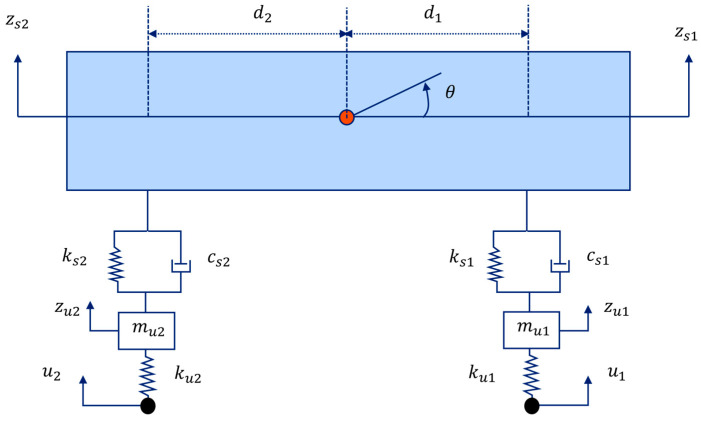
The vehicle system model.

The vehicle model used in this study is a half-car model Figure 2. In this model, $z_{s1}$, $z_{s2}$ are the front and rear sprung-mass vertical accelerations, respectively; $k_{s1}$, $k_{s2}$ are the front and rear spring stiffness coefficients; $c_{s1}$, $c_{s2}$ are the front and rear damping coefficients; $k_{u1}$, $k_{u2}$ are the front and rear unsprung stiffness coefficients; $u_{1}$, $u_{2}$ are the front and rear axle input profiles; and θ is the pitch angle of the vehicle body. The equation of motion of this vehicle model can be written as follows:

(3) $$M_{v}\ddot{z}\left(t\right)+C_{v}\dot{z}\left(t\right)+K_{v}z\left(t\right)=f_{v}\left(t\right)$$

where $M_{v}$ is the mass matrix, $C_{v}$ is the damping matrix, $K_{v}$ is the stiffness matrix for the vehicle system, and $f_{v}\left(t\right)$ is the restoring force due to the input profile. If $m_{s}$ is the vehicle body mass, then $m_{s1}=\frac{d_{2}m_{s}}{d_{1}+d_{2}}$ and $m_{s2}=\frac{d_{1}m_{s}}{d_{1}+d_{2}}$. The components of these matrices and vectors are shown as follows:

(4) $$M_{v}=\left[\begin{array}{cc} \begin{array}{cc} m_{s1} & 0\\ 0 & m_{s2} \end{array} & O\\ O & \begin{array}{cc} m_{u1} & 0\\ 0 & m_{u2} \end{array} \end{array}\right]$$

$$C_{v}=\left[\begin{array}{cccc} c_{s1} & 0 & {-c}_{s1} & 0\\ 0 & c_{s2} & 0 & -c_{s2}\\ -c_{s1} & 0 & c_{s1} & 0\\ 0 & -c_{s2} & 0 & c_{s2} \end{array}\right]$$

$$K_{v}=\left[\begin{array}{cccc} k_{s1} & 0 & -k_{s1} & 0\\ 0 & k_{s2} & 0 & -k_{s2}\\ -k_{s1} & 0 & k_{s1}+k_{u1} & 0\\ 0 & -k_{s2} & 0 & k_{s2}+k_{u2} \end{array}\right]$$

$$z\left(t\right)=\left(\begin{array}{c} z_{s1}\left(t\right)\\ z_{s2}\left(t\right)\\ z_{u1}\left(t\right)\\ z_{u2}\left(t\right) \end{array}\right),\;f_{v}\left(t\right)=\left(\begin{array}{c} 0\\ 0\\ k_{u1}u_{1}\left(t\right)\\ k_{u2}u_{2}\left(t\right) \end{array}\right)$$

where $u_{i}\left(t\right)$ is i-th input profile.

#### 2.1.3. Interactions

(a) Contact forces

The contact force vector $f_{b}\left(t\right)$, which is the input to the bridge system, is calculated as an equivalent nodal force. Details on how to calculate the nodal contact force vector are given here [34].

(b) Input profiles

The inputs to the vehicle’s equation of motion consist of road unevenness (road profile) and bridge deflections. In the numerical simulation, the road profile is given by the following equation [35,36]:

(5) $$r\left(x\right)=\sum_{\xi =1}^{N}\sqrt{\Phi \left(\Omega_{\xi }\right)\Delta \Omega \pi }\sin\;\left(\Omega_{\xi }x-\phi_{\xi }\right)$$

where $\phi_{\xi }$ are the random phase angles, $\Omega_{\xi }$ is the angular spatial frequency, and $\Phi (\Omega_{i})$ is the power spectral density of the road unevenness. The input profile $u_{i}\left(t\right)$ is given by the following formula [30]:

(6) $$u_{i}\left(x\right)=r\left(x_{i}\left(t\right)\right)+y(x_{i}\left(t\right),t)$$

## 3. The Proposed Method

### 3.1. The Basic Theory of the Proposed Method

Equation (2) is used in numerical simulations to calculate bridge vibrations at any given location x and time t. However, in practical scenarios, measuring bridge vibrations at each node is challenging. A simpler equation that correlates the bridge vibrations at sensor locations with another measurement, vehicle vibrations, is preferable.

Therefore, this study proposes a monitoring scheme based on modal analysis theory [8]. By applying modal analysis theory to Equation (1), the following equation is derived:

(7) $${\ddot{q}}_{k}\left(t\right)+\omega_{k}^{2}q_{k}\left(t\right)=\sum_{i}^{n}\frac{m_{i}}{M}\phi_{k}\left(x_{i}\left(t\right)\right)s_{vi}\left(t\right)$$

$$\omega_{k}^{2}={\left(\frac{k\pi }{L}\right)}^{4}\frac{EI}{\rho A}$$

where $q_{k}\left(t\right)$ is the k-th order modal response of the bridge, $\omega_{k}$ is the angular natural frequency of the bridge, $m_{i}$ is the vehicle mass, M=ρAL/2, $\phi_{k}\left(x_{i}\left(t\right)\right)$ is the window function based on k-th mode shape of the bridge, and $s_{i}$ is the sensor measurement of vehicle vibration given by the following equation:

(8) $$s_{vi}(t)=\left(g-{\ddot{z}}_{i}\left(t\right)\right)$$

This approach simplifies the relationship between the bridge and vehicle vibrations, making it more feasible for practical applications in SHM. The derived equation assumes that bridge vibrations, vehicle vibration, and position are simultaneously measured.

The proposed method uses the Frequency Response Function (FRF) to identify the natural frequencies of the bridge structure. The possibility of simultaneously monitoring the vehicle and bridge dynamic response is explored, unlike the existing method that uses only the bridge’s dynamic response. By performing a modal analysis of the bridge’s frequency response due to the vehicle’s excitation, the modal frequencies of the bridge can be identified. The vertical displacement of the bridge $y\left(x,t\right)$, in Equation (2), is expressed in terms of modal coordinates by Equation (9).

(9) $$s_{bi}(t)=\ddot{y}\left(X_{i},t\right)=\sum_{k=1}^{n_{b}}\phi_{k}\left(X_{i}\right){\ddot{q}}_{k}\left(t\right)$$

where $s_{bi}(t)$ is the measurement of i-th sensor on the bridge, $q_{k}\left(t\right)$ is the k-th order basis coordinate, k is the modal number, and $\phi_{k}\left(x\right)$ is k-th mode shape. $\phi_{k}\left(x\right)$ is also of a sinusoidal form given by Equation (7).

(10) $$A_{ik}=\sum_{k=1}^{n_{b}}\phi_{k}\left(X_{i}\right)=\sum_{k=1}^{n_{b}}\sin\;\left(\frac{k\pi X_{i}}{L}\right)$$

where $X_{i}$ is the measurement point on the bridge (i-th sensor’s location), and L is the bridge span length. In this formula, x=0 indicates the entrance of the bridge. This process assumes that the bridge mode shapes are sine curves.

Then, the bridge modal response $\ddot{q}\left(t\right)$ ($={\left[\ldots {\ddot{q}}_{k}\left(t\right)\ldots \right]}^{T}$) is given by the following Equation (11):

(11) $$\ddot{q}\left(t\right)=A^{-1}s_{b}\left(t\right)$$

where A is the mode matrix, of which the $\left(i,\;k\right)$ component is $A_{ik}$, and $s_{b}\left(t\right)={\left[\ldots s_{bi}\left(t\right)\ldots \right]}^{T}$.

### 3.2. Window Functions and Modal Loads

The window function $\phi_{k}\left(x_{i}\left(t\right)\right)$ helps to accurately extract the portion of vehicle vibration data when the vehicle crosses the bridge. This function is given as a sinusoidal function by Equation (12).

(12) $$\phi_{k}\left(x_{i}\left(t\right)\right)=\sum_{k=1}^{n}\sin\;\left(\frac{n_{k}\pi x_{i}\left(t\right)}{L}\right)$$

where k is the modal number, L is the bridge span length and $x_{i}\left(t\right)$ corresponds to the front and rear wheel position over time. On the other hand, the modal load is calculated as a product of the window function and the sensor measurement of vehicle vibration given in Equation (9). Thus, the modal load is given by Equation (13).

(13) $$f_{k}\left(t\right)=\sum_{i}^{n}\phi_{k}\left(x_{i}\left(t\right)\right)s_{vi}\left(t\right)$$

The influence of M in Equation (7) is ignored but it does not affect the accuracy of estimating bridge natural frequencies.

### 3.3. Procedure of the Proposed Method

**Figure 3:**
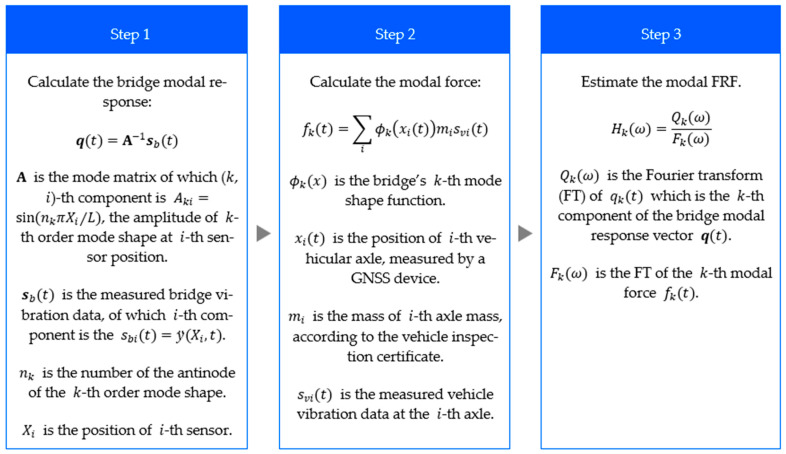
The procedure for simultaneous monitoring.

The proposed method procedure is described in the following diagram. After the acceleration data for both the bridge and vehicle system are obtained, the bridge modal response and modal contact force are calculated from the formulae given in the diagram Figure 3 and then the modal FRF is estimated as the ratio of modal response to modal contact force in the frequency domain.

## 4. Numerical Simulation

### 4.1. VBI Simulation

While the VBI system is nonlinear, it is possible to simulate vehicle and bridge vibrations by iteratively, solving the vehicle system and bridge system separately using direct integration schemes such as Newmark-β or Wilson-θ. For a more comprehensive understanding of this approach based on Equations (2) and (3), readers should refer to the detailed procedure in [33,34].

### 4.2. VBI Models

The system parameters of the vehicle and bridge models are shown in Table 1 and Table 2 respectively. In this study, a half-car model of 13,560 kg body mass is used. On the other hand, the bridge model is assumed to be a Euler–Bernoulli beam of varying parameters.

The bridge parameters are varied to give insight into the performance of the proposed method to bridges of different span lengths, weights, flexural rigidity, and so on. In the simulation, four configurations of bridge spans, medium (40 m) and small (20 m), that are easily excited by vehicle vibrations, are prepared. Different vehicles and different bridges are expected to produce entirely different complex stochastic processes. However, the modal FRFs, under ideal conditions, should theoretically be the same. The system parameters are selected with reference to previous publications with modification [37]. The road bridge specified by Okabayashi and Yamaguchi [37] has span lengths of 20 m to 70 m, and the bridge parameters are adopted according to the design specifications in Japan. The compiled bridge models are presented in Table 2.

The damping coefficients α and β are the coefficients of Rayleigh damping for the mass matrix and stiffness matrix, respectively. The damping matrix is C=αM+βK. The natural frequencies of the vehicle and bridge models are also shown in Table 3.

### 4.3. Simulated Signals

This section presents the simulated vehicle and bridge vibration data for all four bridge models considered. In this section, note that the vibration data of sprung and unsprung mass are used separately to compare how they affect the accuracy of estimating bridge natural frequencies.

**Figure 4:**
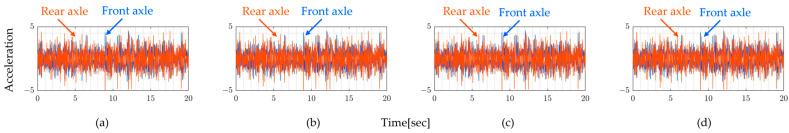
Vehicle’s sprung-mass acceleration vibration for the 4 bridge models shown in Table 2: (**a**) Model 1, (**b**) Model 2, (**c**) Model 3, and (**d**) Model 4.

Figure 4 shows the numerically simulated vibration data of the vehicle’s sprung mass $m_{s1}$ and $m_{s2}$. According to this figure, it is observed that the sprung-mass accelerations remain unchanged, even when the bridge model is altered. This phenomenon can be attributed to the dominance of road profiles affecting vehicle vibrations. In this simulation, the same road unevenness is assumed throughout.

From Table 4, it is evident that the dominant frequencies of the sprung vibration do not coincide with the natural frequencies of the bridge. Additionally, different peaks are observed for the front and rear wheels. This suggests that identifying the natural frequencies of the bridge from the vehicle’s sprung vibrations is challenging. The variations in vibration frequencies between the front and rear wheels further complicate this task, indicating that the vehicle’s response does not straightforwardly reflect the bridge’s natural frequencies. This finding underscores the complexity of extracting bridge characteristics from vehicle vibrations within the VBI system.

**Figure 5:**
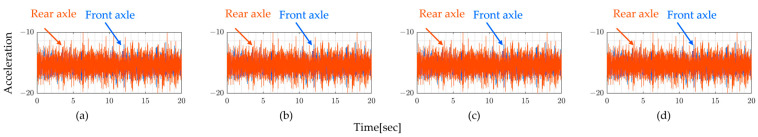
Vehicle’s unsprung-mass acceleration for the 4 bridge models shown in Table 2: (**a**) Model 1, (**b**) Model 2, (**c**) Model 3, and (**d**) Model 4.

Figure 5 similarly displays numerically simulated data of vehicle unsprung-mass vibrations $m_{u1}$ and $m_{u2}$. These figures also illustrate that identifying the bridge’s natural frequencies from the dominant frequencies of these unsprung-mass vibrations is still challenging Table 5. Usually, VBI systems are not significantly affected by the frequency characteristics of bridges, but the road profiles.

**Figure 6:**
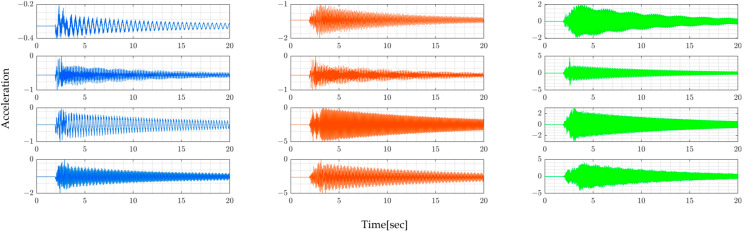
Bridge: (blue) Bridge acceleration at L/4, (red) midspan L/2, and (green) 3L/4, for 4 bridge models shown in Table 2.

On the other hand, three bridge sensors are simulated installed on the bridge. They collect bridge vibration on fixed points that are equally spaced, as shown in Figure 6.

**Figure 7:**
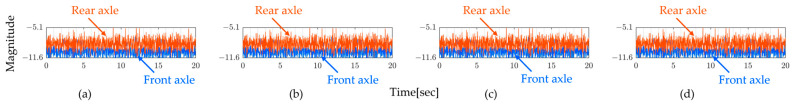
Vehicle contact forces from sprung mass for the 4 bridge models shown in Table 2: (**a**) Model 1, (**b**) Model 2, (**c**) Model 3, and (**d**) Model 4.

To calculate the contact forces shown in Figure 7, both sprung-mass and unsprung-mass vertical vibration are considered. Even though the bridge models are varied, the contact force characteristics are not changed. This means that the influence of bridge deflection in vehicle responses is small enough relative to the road unevenness.

### 4.4. Numerical Results and Discussion

**Figure 8:**
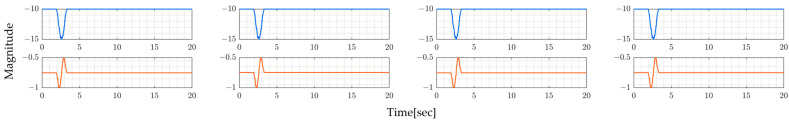
Bridge modal forces, (blue) front axle, (red) rear axle, for the 4 bridge models shown in Table 2.

The modal contact force calculated from Equation (13) is shown in Figure 8. It can be shown that the force magnitude is zero when the vehicle is outside the bridge span. Note that the mass of the vehicle is ignored in this scheme because the magnitude of the modal contact force itself does not affect the result.

**Figure 9:**
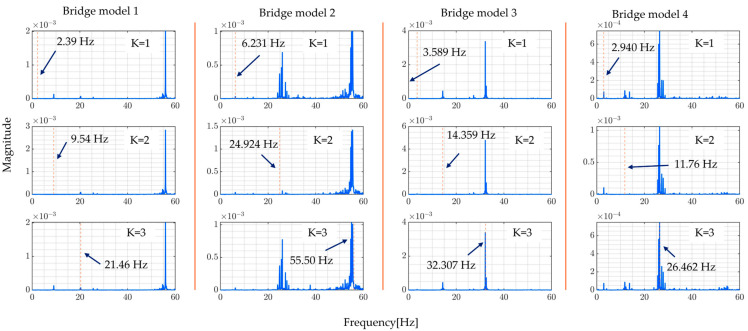
Directly calculated ratio of Fourier transforms of vehicle and bridge responses.

**Figure 10:**
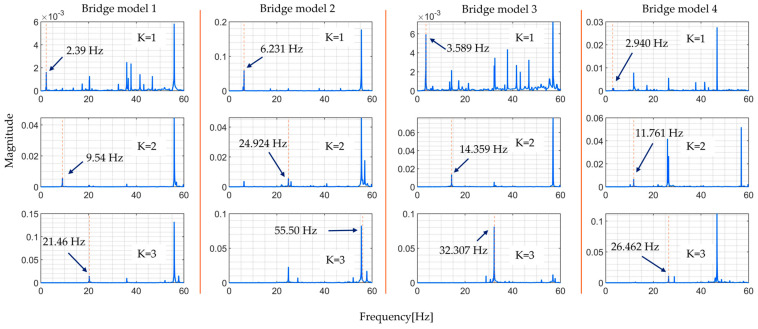
FRFs estimated by proposed method.

The following Figure 9 shows the FRFs estimated by numerical experiments obtained by directly calculating the ratio of Fourier transforms of vehicle and bridge responses, while Figure 10 presents the modal FRFs obtained from the proposed method; the red vertical dashed line indicates where the bridge’s natural frequency is expected to be found.

From these figures, the proposed method can identify higher bridge natural frequencies up to the third mode accurately. However, it is not possible to accurately locate the natural frequencies using the non-modal based method and it is difficult in the case of direct calculation of the ratio of Fourier transforms of vehicle and bridge responses. Different studies explain that this is because higher modes of vibration are not excited and thus cannot be identified by normal treatment [6,20]. This justifies, numerically, the aim of this study which is to improve the accuracy of estimating higher mode of vibration to facilitate the identification of localized bridge damage. Table 3 shows the targeted bridge mode of vibration. They are the values of the natural frequencies of the bridge model used in the numerical simulation.

These results demonstrate the feasibility of the application of modal FRFs for estimating bridge natural frequencies from simultaneous monitoring. Environmental factors such as changes in temperature and noise affect the bridge’s natural frequency estimation accuracy. However, it should be noted that these factors are not considered during the numerical simulation. This will be considered in the future work where environmental noise will be added to the numerical simulated measured data to mimic real bridge environment conditions.

## 5. Field Experiment

### 5.1. Experimental Preparation and Setting

The experiment was carried out on a 30.82 m span bridge, using a 10-ton truck. During the field experiment with the truck, a multipoint data measurement was performed. The vibration measurement system consisted of ZYBOZynq-7010 (manufactured by XILINX., San Jose, CA, USA) used as an FPGA (Field Programmable Gate Array) board equipped with an ARM processor which is CORTEX-A9 (manufactured by Arm Holdings plc., Cambridge, UK) and an expansion board equipped with an accelerometer module and a GNSS receiver. The ADXL355 (manufactured by Analog Devices Inc., Norwood, MA, USA) was used as a three-axis accelerometer module. This module has a 20-bit ADC resolution and ±8 G range. The GNSS receiver used was AE-GYSFDMAXB (manufactured by Taiyo Yuden, Tokyo, Japan). The sampling rate for time, position information, and acceleration vibration data was 300 Hz. Since the GNSS receiver receives satellite signals at 1Hz, it is converted to 300 Hz by interpolation. Time synchronization between vehicles and bridges was corrected by referring to the acquisition time of the PPS (Pulse Per Second) signal from the GNSS receiver.

**Figure 11:**
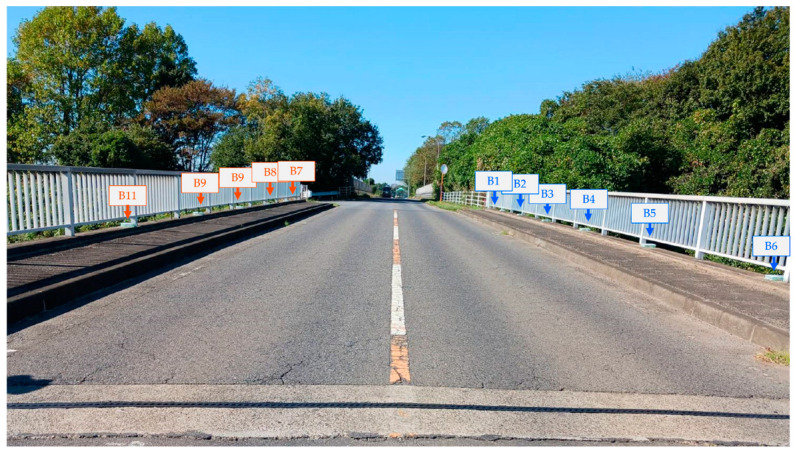
The monitored bridge.

**Figure 12:**
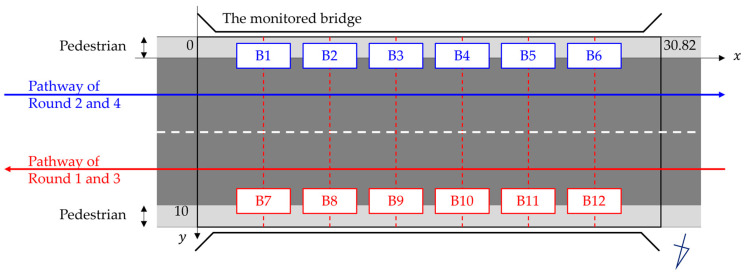
Sensor installation and vehicle pathways.

A total of 12 bridge sensors were installed along the sidewalk of the monitored bridge, 6 sensors on each side separated by approximately 4.3 m, and were named B1 to B12, as is shown in Figure 11 and Figure 12. The sensor system consisted of accelerometers and GPS sensors. The GPS sensors were installed on the truck and helped in temporal synchronization and vehicle location tracking with respect to the monitored bridge.

### 5.2. Obtained Data

**Figure 13:**
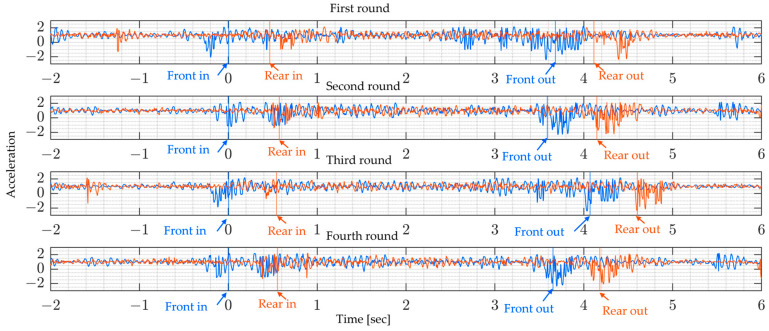
Vehicle vibration on bridge for four runs: (blue) front axle, and (red) rear axle.

**Figure 14:**
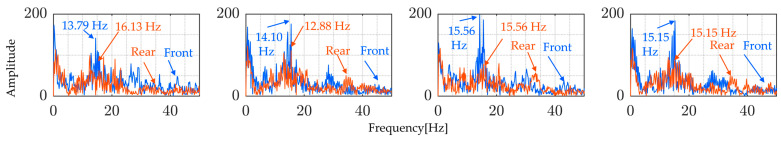
Vehicle-measured vibration spectrum for four runs: (blue) front axle, (red) rear axle.

The sampling rate of the sensor used is 330 Hz. The following figures present the measurement signals which show 1 G (gravitational acceleration) data upward shift due to the sensor settings. Figure 13 shows the vehicle’s measured vertical acceleration for the front and rear axle, and Figure 14 shows the frequency content of the measured vehicle’s vertical acceleration of all four rounds for 6 s duration.

**Figure 15:**
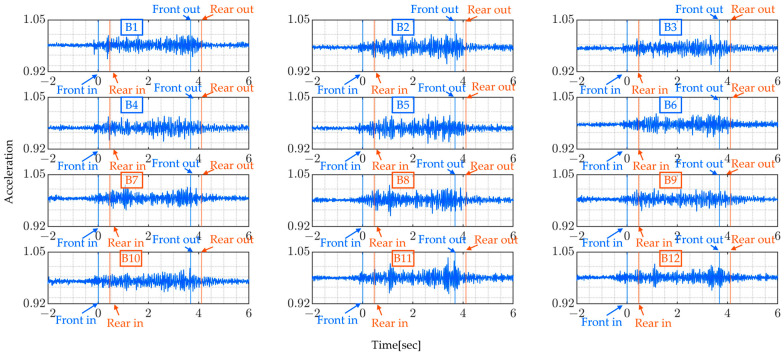
Bridge vibration data ${\ddot{y}}_{i}(t)$ (Round 1).

From Figure 14, it can be shown that the vehicle’s predominant frequencies do not vary much, especially since the last two rounds show the same frequencies for both the rear and front axles. The following Figure 15 shows the bridge’s vertical acceleration vibrations. As expected, the bridge vibrations were around free vibration before and after the monitored truck entered the bridge. The vibration recorded before might be due to the presence of other vehicles on the bridge and similarly, after the truck has left the bridge.

The added red and blue lines indicate when the vehicle enters and leaves the bridge span. This is made possible using GNSS devices mounted on the vehicle. In this figure, the bridge vibration shows a similar trend at all measured points, which suggests that it is not possible to identify bridge vibration characteristics by simply looking at acceleration signals.

Table 6 summarizes the dominant frequencies from the power spectrum of measured bridge vibration data. The highest, in the range of 0−50 Hz frequencies, varies between 12.07 Hz and 17.41 Hz. These figures reveal that all the bridge vibration data have the same trends at different frequencies across all rounds. The presence of distinctive frequency peaks for both lower and higher frequencies suggest the possibility of identifying the bridge’s natural frequency after the application of the proposed method to the measured data. Furthermore, the predominant frequency may change depending on loading conditions and should not be confused with the bridge’s natural frequencies.

### 5.3. Results and Discussion

**Figure 16:**
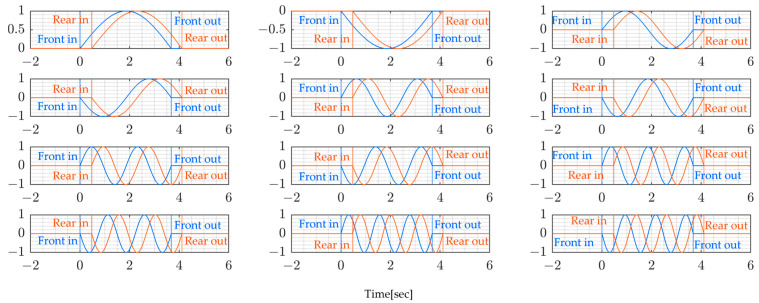
Window function for the paths of the 1st and 3rd rounds.

**Figure 17:**
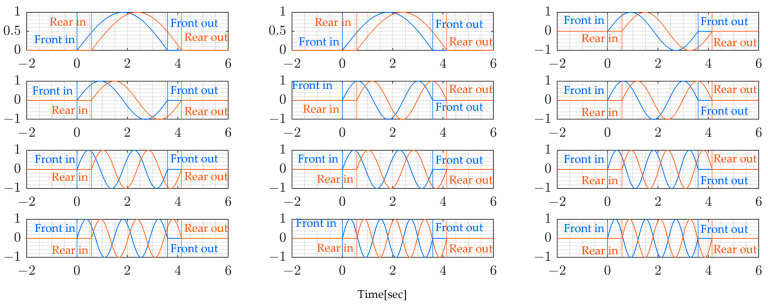
Window function for the paths of the 2nd and 4th rounds.

This section presents and discusses the obtained results. Figure 16 and Figure 17 present the window functions used. Since the bridge was constantly crossed by other vehicles, which were not part of the experiment, the time during which the experiment truck crossed the bridge was extracted and the window function, which is a sinusoidal function, was used to eliminate unwanted signals from other vehicles. This allowed the calculation of modal forces in the time window when the vehicle was on the bridge span length.

**Figure 18:**
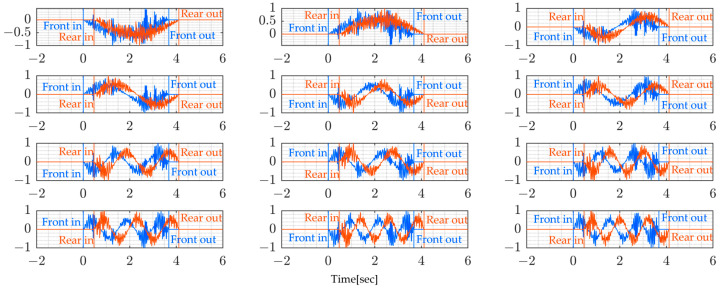
Modal force for the paths of the 1st and 3rd rounds.

**Figure 19:**
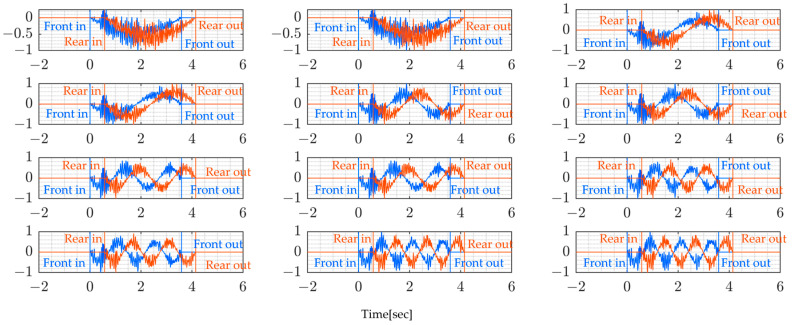
Modal force for the paths of the 2nd and 4th rounds.

The following Figure 18 and Figure 19 present the modal forces which are the inputs to the bridge system. The modal forces are calculated from Equation (13). These forces are essential for the analysis of the dynamic behavior of bridges under the moving vehicle. The modal forces are usually modeled as a series of moving concentrated loads, but under the current analysis, the vehicle position is considered so that the modal force is a time-varying quantity.

**Figure 20:**
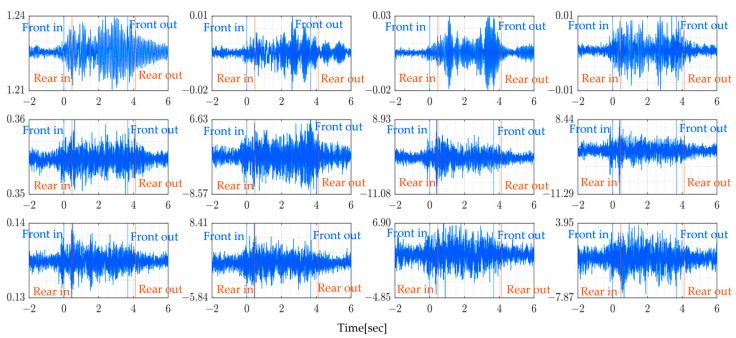
Bridge modal response (Round 1).

After the calculation of modal forces, the bridge modal response can be calculated from Equation (11). Figure 20 presents the calculated bridge modal responses. According to this figure the modes of vibration are very close and difficult to distinguish. Hence the application of frequency analysis methods that do not consider modal analysis will not be effective, which suggests that a modal-based FRF scheme is appropriate in this case.

**Figure 21:**
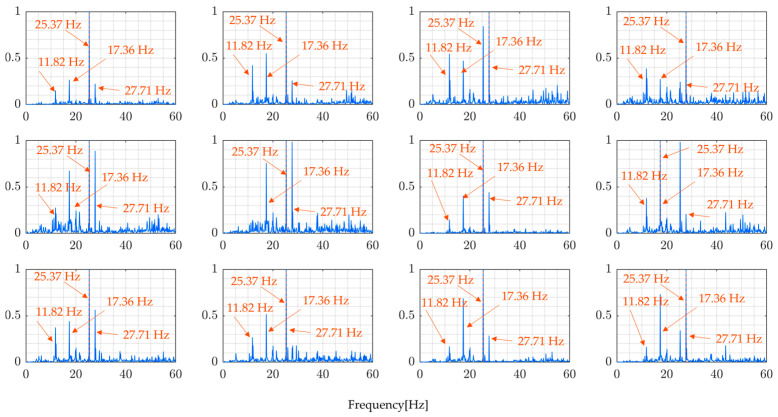
Directly calculated ratio of Fourier transforms of vehicle and bridge responses in the field experiment (Round 1).

**Figure 22:**
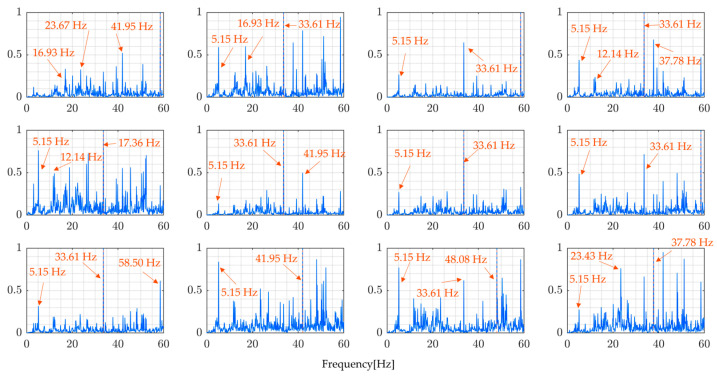
Directly calculated ratio of Fourier transforms of vehicle and bridge responses in the field experiment (Round 2).

Figure 21 and Figure 22, as well as Table 7, present the results obtained by directly calculating the ratio of the Fourier spectra of vehicle vibration and bridge vibration, ignoring the effect of vehicle movement, in other words, vehicle position which changes with time. The FRF shown in these figures is incorrect in that it does not accurately calculate the external force acting on the bridge. Such a process results in a pseudo FRF with many peaks, significantly reducing the accuracy of the estimates of the natural frequencies.

**Figure 23:**
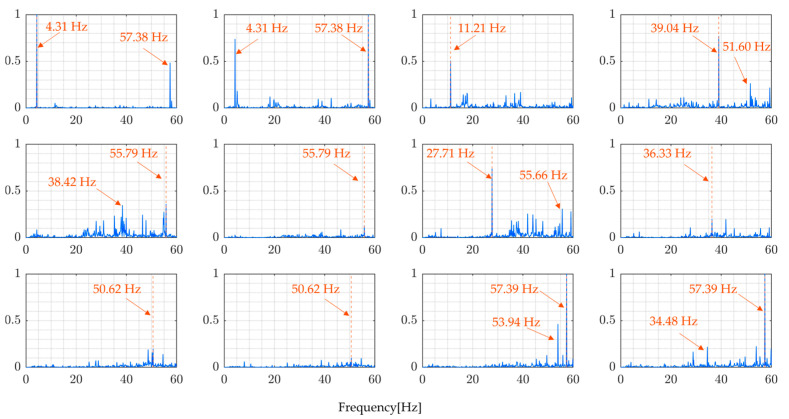
Modal FRFs (Round 1).

**Figure 24:**
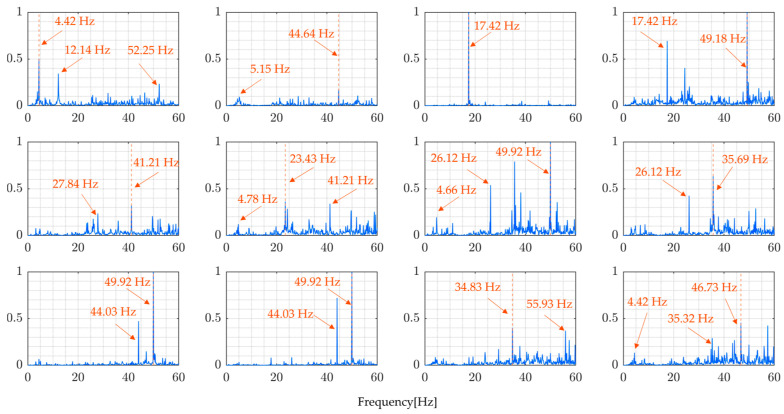
Modal FRFs (Round 2).

The modal FRFs estimated by the proposed method, for all four rounds of the experiment, are presented in Figure 23 and Figure 24. These figures clearly show identifiable frequency peaks, suggesting that the proposed method can identify the higher modes of vibration of the bridge. The identification of frequency peaks is essential for the frequency characterization of the bridge vibration modes. The ability to identify the higher modes of vibration that are sensitive to localized bridge damage helps in understanding the bridge’s dynamic characteristics and informing SHM and maintenance efforts. The distinguishable frequency peaks in the estimated modal FRFs demonstrate the potential of the proposed method for modal identification and analysis.

The modal FRFs estimated by the proposed method, even though incomplete, showed a reduction in the number of peaks in each FRF graph. Ideally, each modal FRF is expected to have a single peak. Bridges are complex structures, and it has been confirmed that fully estimating modal FRFs is challenging. In addition, the process of estimating modal FRFs used in the proposed method involves strong assumptions, such as treating the mode shapes as sine curves. In the future, by modifying these assumptions, it is conceivable that more plausible modal FRFs can be estimated.

Comparing the results of the proposed method, Figure 23 and Figure 24, and the results from directly calculating the ratio of the Fourier spectra of vehicle vibration and bridge vibration, Figure 21 and Figure 22, the implications of the above results become clear. In simultaneous vibration measurements, it is necessary to measure the relative position of the vehicle on the bridge at the time. Furthermore, it is important to estimate natural frequencies to properly consider the changes in traffic load due to the vehicle’s position based on modal analysis theory and to determine the FRFs accordingly.

Even though the frequency peaks are clearly identifiable, it is still difficult to confirm whether the identified frequencies shown in Table 8 are the natural frequencies of the monitored bridge. The modal parameters need to be compared with the basis values to confirm their accuracy. The basis values can be the vibration mode values recorded before the bridge is open to traffic or the value from a free-vibration test conducted with precision. In this case, there were no basis values for the vibration mode of the monitored bridge, which made it difficult to confirm the accuracy of the proposed method using field experimental data.

Each modal FRF is supposed to have a single peak. However, the estimated modal FRFs have multiple peaks, suggesting several technical challenges remain, including assumptions about mode shapes being sine curves, noise, and errors in position measurement. Nevertheless, the fact that we could demonstrate the feasibility of calculating modal FRFs is significant, offering new options for bridge maintenance. Future research should focus on developing the method further by using different vehicles and combining bridge vibrations with vibrations from each vehicle, to reliably identify the same FRFs.

## 6. Conclusions

This paper proposes a new FRF-based simultaneous monitoring method that aims to enhance the accuracy of estimations of a bridge’s higher-order modal frequencies by utilizing a GNSS device for time synchronization. The proposed method was tested by numerical simulations and field experiments. The numerical simulation results show that the proposed method is effective in the estimation of bridge modal frequencies up to the third mode. Contrary to the existing method, which does not consider modal analysis, it is difficult to identify the bridge modal frequency.

On the other hand, the results from the application of the proposed method to the field experimental data clearly show distinct frequency peaks for each set of sensor data analyzed. Unfortunately, it is not easy to confirm that the identified frequency peaks correspond to the monitored bridge’s modal frequencies. The reason is that the basis value, or in other words, the value for the intact case, was not available for the monitored bridge. The use of GNSS for time synchronization and simultaneous monitoring presents an innovative direction in bridge structure health monitoring and damage detection.

However, it is crucial to note that simultaneous vibration measurement using GNSS is only effective under conditions where traffic vibration is predominant. That is, in cases like long-span bridges where wind loads are significantly greater than traffic loads, the proposed method is not necessary. In such cases, bridge vibrations should exhibit peaks near their natural frequencies due to random white noise input, allowing for direct estimation of the bridge’s natural frequencies from the measurements of bridge vibrations. However, for short- and medium-span bridges, where traffic vibrations are predominant, it is necessary to estimate higher-order modes. In such cases, estimating modal FRFs is important, and now that the vehicle and bridge vibrations can be measured simultaneously, this issue will be solved. Unfortunately, the experimental results suggest that the modal FRFs were not calculated correctly, likely due to the bridge being more complex than anticipated. Some of the assumptions made may need to be reconsidered more cautiously. This study, as an initial step, has demonstrated that the scheme for estimating modal FRFs is feasible. In the future, to reliably and accurately estimate modal FRFs, more advanced schemes, such as identification using multiple vehicles, need to be developed.

## Figures and Tables

**Table 1:** Vehicle system parameters.

Parameter Name	Symbol	Value	SI Unit
Body mass	$m_{s}$	13,560	kg
Unsprung mass (front)	$m_{u1}$	751	kg
Unsprung mass (rear)	$m_{u2}$	469	kg
Suspension stiffness (front)	$k_{s1}$	456 × 10^3^	N/m
Suspension stiffness (rear)	$k_{s2}$	41.0 × 10^4^	N/m
Suspension damping (front)	$c_{s1}$	29.0 × 10^3^	Ns/m
Suspension damping (rear)	$c_{s2}$	24.2 × 10^3^	Ns/m
Tire stiffness (front)	$k_{u1}$	431 × 10^4^	N/m
Tire stiffness (rear)	$k_{u2}$	431 × 10^4^	N/m
Distance from the front axle to the center of gravity	$d_{1}$	2.00	m
Distance from the rear axle to the center of gravity	$d_{2}$	2.40	m
Velocity	v	10.0	m/s

**Table 2:** Bridge model system parameters.

Bridge Model	Flexural Rigidity EI [N/m^2^]	Mass Per Unit Length ρA [kg/m]	Span Length L [m]	Damping Coefficient 1 α	Damping Coefficient 2 β
Model 1	12.369 × 10^9^	6624	30	1.2 × 10^−1^	1.0 × 10^−6^
Model 2	6.0850 × 10^9^	2417	20	1.2 × 10^−1^	1.0 × 10^−6^
Model 3	12.170 × 10^9^	2583	30	1.2 × 10^−1^	1.0 × 10^−6^
Model 4	23.920 × 10^9^	2667	40	1.2 × 10^−1^	1.0 × 10^−6^

**Table 3:** The natural frequencies of the vehicle and bridge models.

	First Mode (Hz)	Second Mode (Hz)	Third Mode (Hz)	Fourth Mode (Hz)
Vehicle	1.210	2.549	12.694	15.980
Bridge Model 1	2.259	9.039	20.338	36.156
Bridge Model 2	6.231	24.924	56.078	99.695
Bridge Model 3	3.589	14.359	32.307	57.435
Bridge Model 4	2.940	11.761	26.462	47.044

**Table 4:** Dominant frequencies of the vehicle’s sprung-mass acceleration for the 4 bridge models shown in Table 2.

Axle	Dominant Frequency Order	Bridge Model 1	Bridge Model 2	Bridge Model 3	Bridge Model 4
Front Axle	First	3.4	3.4	3.4	3.4
	Second	8.15	8.15	8.15	8.15
	Third	15.15	15.15	15.15	15.15
	Forth	20.75	20.75	20.75	20.75
Rear Axle	First	2.25	2.25	2.25	2.25
	Second	8.15	8.15	8.15	8.15
	Third	15.15	15.15	15.15	15.15
	Forth	20.75	20.75	20.75	20.75

**Table 5:** Dominant frequencies of the vehicle’s unsprung-mass acceleration for the 4 different bridge models shown in Table 2.

Axle	Dominant Frequency Order	Bridge Model 1	Bridge Model 2	Bridge Model 3	Bridge Model 4
Front Axle	First	10.65	10.65	10.65	10.65
	Second	11.85	11.85	11.85	11.85
	Third	13.37	13.37	13.37	13.37
	Forth	-	-	-	-
Rear Axle	First	15.15	15.15	15.15	15.15
	Second	16.65	16.65	16.65	16.65
	Third	17.25	17.25	17.25	17.25
	Forth	-	-	-	-

**Table 6:** The observed first dominant frequencies of bridge vibration data in each round.

Round	Sensor Number											
	B1	B2	B3	B4	B5	B6	B7	B8	B9	B10	B11	B12
1	17.36	12.07	12.07	12.07	12.07	17.00	17.36	17.36	12.07	12.07	17.36	17.36
2	13.12	13.12	12.26	12.26	12.26	12.26	16.80	16.68	12.26	12.14	12.26	16.80
3	17.41	17.41	12.07	12.07	17.41	17.07	17.41	17.41	11.96	12.07	-	17.41
4	16.98	16.98	16.98	11.97	16.98	16.98	16.98	16.98	11.97	11.97	-	16.98

**Table 7:** The estimated natural frequencies of the direct-calculation method.

Round	Modal Order (k)											
	1	2	3	4	5	6	7	8	9	10	11	12
1	11.82	17.36	25.37	27.71	12.07	-	-	-	-	-	-	-
2	3.07	12.39	16.93	23.67	33.61	-	-	-	-	-	-	-
3	3.02	13.58	26.35	-	-	-	-	-	-	-	-	-
4	7.57	12.34	17.34	21.62	23.70	-	-	-	-	-	-	-

**Table 8:** The estimated natural frequencies of the bridge from modal FRFs.

Round	Modal Order (k)											
	1	2	3	4	5	6	7	8	9	10	11	12
1	4.31	5.17	11.21	14.16	38.42	41.87	49.51	-	-	-	-	-
2	4.42	5.15	11.65	17.42	26.12	35.69	49.92	-	-	-	-	-
3	3.02	5.11	11.84	16.95	37.96	36.80	48.76	-	-	-	-	-
4	4.28	5.01	11.84	17.34	25.16	38.11	46.78	-	-	-	-	-

## Data Availability

Data are contained within the article.
